# Cardiac autonomic disturbance following sprint-interval exercise in untrained young males: Does exercise volume matter?

**DOI:** 10.1016/j.jesf.2021.10.002

**Published:** 2021-10-30

**Authors:** Yingfeng Ye, Tomas K. Tong, Zhaowei Kong, Emma Dan Tao, Xiaohua Ying, Jinlei Nie

**Affiliations:** a National Health Committee Key Lab of Health Technology Assessment (Fudan University), Department of Health Economics, School of Public Health, Fudan University, Shanghai, 200032, China; b Dr. Stephen Hui Research Centre for Physical Recreation and Wellness, Hong Kong Baptist University, Hong Kong; c Faculty of Education, University of Macau, Macao; d College of Physical Education, Liaoning Normal University, Dalian, Liaoning, 116029, China; e School of Health Sciences and Sports, Macao Polytechnic Institute, Macao

**Keywords:** Sprint-interval training, Heart rate variability, Cardiovascular system, Cardiac parasympathetic activity, Cardiac health

## Abstract

**Objectives:**

This study examined the influences of the volume of all-out sprint-interval exercise (SIE) on acute post-exercise heart rate variability (HRV) recovery.

**Methods:**

HRV recovery following a session of (i) 2 × 30-s SIE (SIE_2_), (ii) 4 × 30-s SIE (SIE_4_), and (iii) non-exercising control (CON) were compared in 15 untrained young males. Time domain [standard deviation of normal-to-normal intervals, root mean square of successive R-R differences] and frequency domain [low frequency (0.04–0.14 Hz), high frequency (0.15–0.40 Hz)] measures of HRV were assessed every 20 min for 140 min after the exercise, and every hour during the first 4 h of actual sleep time at immediate night. All trials were scheduled at 19:00.

**Results:**

In comparison to CON, both SIE_2_ and SIE_4_ attenuated the HRV markedly (*p* < 0.05), while the declined HRV restored progressively during recovery. Although the sprint repetitions of SIE_4_ was twice as that of SIE_2_, the declined HRV indices at corresponding time points during recovery were not different between the two trials (*p* > 0.05). Nevertheless, the post-exercise HRV restoration in SIE_2_ appeared to be faster than that in SIE_4_. Regardless, nocturnal HRV measured within 10 h following the exercise was not different among the SIE and CON trials (*p* > 0.05).

**Conclusion:**

Such findings suggest that the exercise volume of the SIE protocol may be a factor affecting the rate of removal of the cardiac autonomic disturbance following the exercise. In addition, rest for ∼10 h following either session of the SIE protocol appears to be appropriate for the cardiovascular system to recover.

## 1 Introduction

The high-intensity interval training that consisted of repeated all-out supramaximal sprints (>100% VO_2max_), with exercise bout of ≤30 s, was termed as sprint-interval training.^1^ Such specific training regimens have been demonstrated effective to induce energetic adaptation and fat loss in both healthy and obese people, and are generally considered as time-efficient non-pharmacological strategies for promoting cardiovascular and metabolic health.^2^, ^3^, ^4^, ^5^

During the all-out sprint-interval exercise (SIE), the work of cardiovascular system increases markedly in order to meet the enormous metabolic demand.^6^ The associated increase in heart rate (HR) from the resting level is mainly accomplished by the autonomic regulation of the heart, i.e. increase in sympathetic modulation and withdrawal of parasympathetic activity of the autonomic nervous system to the heart.^7^ Such exercise-induced imbalance in the autonomic nervous activity, which results in an increase in HR and an associated decrease in heart rate variability (HRV), can sustain post exercise for up to 72 h, depending upon various factors including preceding exercise intensity, age, sex and training status.^8^^,^^9^ The decreased HRV reflects the inadequate regulatory capacity of the body in adaptively respond to challenges like exercise or stressors.^10^ It is also a sign of depletion of energy reserves and pathology.^10^^,^^11^ Previous studies have demonstrated that the time-dependent HRV recovery of individuals after a training session, which reflects the reactivation of the cardiac parasympathetic neural activity, can be used as a cardiovascular system recovery marker in guiding the subsequent training load prescription in avoidance of unnecessary overloading and non-functional overreaching.^12^, ^13^, ^14^ Further, knowledge of this effect is also essential for clinicians in monitoring exercising individuals who are prone to adverse cardiovascular events.^7^

Recent reviews reported that a higher preceding exercise intensity resulted in a slower post-exercise HRV recovery.^7^^,^^15^ Further, the exercise mode that recruited larger muscle mass (i.e. running vs cycling) appeared to have a slower parasympathetic reactivation.^16^ The rate of HRV recovery following specific exercise modality at high intensity was likely to be associated with the amount of anaerobic process participation during the exercise, as well as the subsequent stimulation of the muscle metaboreflex.^17^ Regarding the exercise duration, its effects on the post-exercise HRV recovery, in particular following SIE when controlling for the intensity, have not been investigated thoroughly.^7^ It has been reported that in contrast to the insignificant effect of exercise duration on the HRV recovery following moderate-intensity continuous exercise,^18^ double of the distance covered (1.25 vs 2.5 km) in a high-intensity intermittent run (100% maximal aerobic speed) resulted in a tiny but significant delay in vagal restoration during immediate 60-min recovery phrase.^19^ However, the comparisons of the post-exercise HRV recovery in the previous study did not include the corresponding time course of HRV in a control situation. Moreover, the exercise intensity of maximal aerobic speed was lower than the all-out supramaximal level that was traditionally adopted in sprint-interval training regimens for health promotion purpose.^3^ The aim of this study was to compare the acute HRV recovery following a session of 2 × 30-s all-out sprint-interval exercise (SIE_2_) and that of double number of exercise bouts [4 × 30-s SIE (SIE_4_)] on a cycle ergometer in a randomized controlled manner. For assessing the HRV responses, time and frequency domain measures of HRV were analyzed. The parameters in the time domain were the standard deviation of normal to normal intervals (SDNN), and the root mean square of successive R-R differences (RMSSD).^20^ Frequency domain measures, which were extrapolated through the Fast Fourier Transformation algorithm, were the low frequency (LF; 0.04–0.14 Hz), high frequency (HF; 0.15–0.40 Hz), as well as LF/HF ratio.^20^ Since parasympathetic activity is high during night sleep, nocturnal HRV may allow better discrimination of the changes in the equilibrium of autonomic nervous activities.^21^ Nocturnal HRV subsequent to the experimental trials were also compared. It was hypothesized that both the SIE_2_ and SIE_4_ would attenuate the acute post-exercise HRV and nocturnal HRV in comparison to those of non-exercising control (CON) trial, while the attenuations in the SIE_2_ would be less, and the rate of recovery of the attenuated HRV would be faster in comparison to that of SIE_4_.

## 2 Methods

### 2.1 Participants

An a priori, two-tailed power calculation at an alpha of 0.05 and a power of 80%, carried out based on a previous study comparing HRV responses in 30-s all-out sprint-interval session and control session,^22^ suggested that a minimum of 15 participants were required in this study. Accordingly, 15 young male participants (Table 1), who were non-athletes but occasionally participating in recreational sports activities such as basketball or badminton, volunteered to participate in the study. None of the participants were clinically diagnosed with chronic diseases, such as cardiovascular, metabolic, or musculoskeletal disorders. Smokers and those on medication or dietary supplements of various types were excluded from the study. After being fully informed of the experimental procedures and possible discomfort associated with the exercise test, participants gave their written informed consent. Ethical approval for this study was obtained from the College Ethical Committee of Macao Polytechnic Institute. The study was conducted in accordance with the Declaration of Helsinki.

**Table 1:** Physical characteristics of the participants.

Variables	Mean ± SD (*n* = 15)
Age (year)	22.7 ± 2.5
Body height (cm)	173.7 ± 7.3
Body mass (kg)	63.5 ± 6.9
Body mass index (kg.m^−2^)	21.0 ± 1.5
Percentage body fat (%)	16.9 ± 3.0
Peak oxygen uptake (ml.kg^−1^.min^−1^)	39.5 ± 7.5

*Mean ± SD* mean ± standard deviation.

### 2.2 Research design

Following the preliminary tests, all participants performed SIE_2_, SIE_4_, and CON trials. The order in which the three trials were performed were assigned to the participants in a random fashion. In SIE trials, pre- and post-exercise HRV and blood pressure (BP), as well as the nocturnal HRV and BP at the immediate night sleep were measured. In CON trial, the experimental procedures were same as that of the SIE_4_ trial, except that the SIE was replaced by quiet sitting. The HRV and BP measured at corresponding time points under paced respiratory rate were compared among the three trials.

All trials were separated by a minimum of one week to avoid potential training effects. Moreover, all tests were scheduled at the same time of day of 19:00 in avoidance of serious deviation in the time between end of exercise and nocturnal HRV measurements. All participants were instructed to maintain their dietary habits during the study period and to avoid taking any additional nutritional supplements prior to the test. Prior to each trial, the participants refrained from eating for at least 2 h, and from participation in strenuous physical activity for at least one day.

### 2.3 Procedures

#### 2.3.1 Preliminary tests and familiarization trials

Body height was measured using a wall mounted stadiometer (Novel, Illinois, US). Body mass, body mass index, and percentage of body fat were measured by the leg-to-leg bioimpedance measuring system (Inbody 720, Seoul, Korea). For the V̇O_2peak_ measurement, participants exercised on a cycle ergometer at an initial work rate of 60 W and pedal frequency of 60 rpm; power output was increased by 25 W every 3 min until volitional exhaustion. V̇O_2_ was measured using the MetaMax 3B-CPET equipment (Cortex, Leipzig, Germany). V̇O_2peak_ was the highest 15-s average value. Following preliminary testing, participants were familiarized with the control of respiratory rate by following an electronic metronome, and the sprint-interval protocol on a cycle ergometer. This familiarization period introduced the testing equipment and protocols and provided the participants with experience of exercising at all-out intensity.

#### 2.3.2 Experimental trials

SIE_2_ trial was comprised of two 30-s all-out sprints on a stationary cycle ergometer (Monark 839E, Varberg, Sweden) with loading set at 7.5% of the participants’ body mass. The 30-s sprint bouts were interspersed with 4-min self-paced, minimum-load cycling exercise. The session duration of SIE_2_ was 5 min. In SIE_4_ trial, the 30-s sprint bouts and recovery intervals were identical to that of SIE_2_ while the participants repeated the all-out sprint for four times, with the session duration extended to 14 min. All participants were reminded to make an all-out effort in each 30-s sprint bout and avoid any pacing strategy in repeating the sprints during each trial.

For each 30-s sprint bout, participants started cycling at 1 kp for 5 s. At the last 2 s, they began to accelerate while the load was being increased to prescribed level. In the subsequent 30-s sprint, the number of pedal revolutions for each 5-s period was recorded. The peak power, mean power, and the fatigue index defined as the percent decrease from the highest to lowest power were calculated accordingly.

Before the SIE, participants performed a standardized warm-up of 3-min submaximal cycling exercise that was interspersed by all-out sprints at the last 5 s of each minute. The initial loading of the 5-s sprint was set 1 kp below the prescribed level for the SIE test. The load was increased 0.5 kp in each sprint, so that by the last sprint the prescribed value was reached. After the SIE, participants performed 5-min unorganized activities including static stretching for cool down purpose.

### 2.4 Measurements

#### 2.4.1 Pre- and post-exercise HRV

For the pre-exercise HRV measurement, the electrocardiogram (ECG) of participants were recorded using a combined custo screen 200 ABPM and Holter analysis unit (custo med, Ottobrun, Germany) in a quiet laboratory environment with controlled settings (21.2 °C, 66.9% relative humidity). Five electrodes were applied on the chest following the instruction of the custo Holter operation manual. Leads were connected from the electrodes to the Holter adaptor of the combined custo screen 200 unit, which was fixed at the hip, to record the ECG at the sampling rate of 1 KHz. The recording period took place in the supine position for 15 min and participants were instructed to remain awake and silent. The first 10 min was a period for stabilization and necessary adjustment. ECG recorded in the last 5 min were for analysis. Following the pre-exercise measurement, the participants performed the warm-up and SIE protocols, and remained quiescent post exercise after necessary cool down and wash up. For assessing the post-exercise HRV recovery, participants returned to supine position. ECG in the last 7 min of every 20 min was measured until 140 min after the exercise completed. The data of the first and the last minute were discarded, the 5-min segments of ECG starting from 2^nd^ min were extracted for analysis. This time frame of 5 min has been shown sufficient to analyze the data for assessing HRV.^23^ For minimizing the influences of respiration on the pre- and post-exercise HRV measurements, the respiratory rate of participants was controlled throughout the recording periods by following an electronic metronome set at 12 breaths.min^−1^.

For HRV analysis, the ECG data were downloaded to computer through the custo diagnostic software (Version 4.12, custo med, Ottobrun, Germany). Each ECG segment were manually filtered by visual inspection to detect the non-sinus R-R intervals (i.e. ectopic beats). To generate normal-to-normal sinus interval time series, ectopic beats and artifacts were replaced by interpolating adjacent beats. Time and frequency domain measures of HRV were analyzed using Kubios software (Version 3.0, Kuopio, Finland).

For assessing the post-exercise nocturnal HRV in the experimental trials, the same custo screen Holter (custo med, Ottobrun, Germany) was used. The ECG data collected in each 60 min of the first 4 h of actual sleep time of participants were analyzed in term of identical variables of cardiac autonomic activity. The actual sleep time period was monitored by placing an actigraphy of SenseWear armband (SWA Pro3, BodyMedia, PA, US) on the upper non-dominant arm over the triceps during sleep. The estimates of sleep and wake parameters were extracted using the SenseWear software (Professional 7.0).

#### 2.4.2 Pre- and post-exercise BP

Pre- and post-exercise BP, as well as the nocturnal BP, were measured using the same combined custo screen 200 ABPM and Holter analysis unit (custo med, Ottobrun, Germany) every 20 min starting from the beginning of each trial for at most 24 h. The BP cuff was placed on the non-dominant upper arm, 2–3 cm above the crook of the arm. The cuff tube was laid from the shoulder of the measured arm over the other shoulder and connected to the custo screen 200 ABPM monitor of the combined unit which was fixed at the hip. The BP data were downloaded to computer through the custo diagnostic software (Version 4.12, custo med, Ottobrun, Germany), and the data collected from the periods corresponding to that of the ECG measurement were analyzed.

### 2.5 Data analyses

In order to reduce bias arising from skewed distributions and simplify its analysis, all the HRV variables were log-transformed (LnSDNN, LnRMSSD, LnLF, LnHF). Shapiro-Wilk normality test revealed that the data for all variables were normally distributed. The alterations in SIE variables among different bouts in SIE_4_, and their interaction in the 1^st^ and 2^nd^ bouts across SIE_2_ and SIE_4_ were examined using one-way, and two-way ANOVA with repeated measures, respectively. To assess the differences in HRV and BP variables among trials, and across time points, 3 × 8 (Pre- & Post-exercise) and 3 × 4 (nocturnal) repeated measures ANOVA were computed. Post hoc analyses for ANOVA, using the Bonferroni test for identifying simple main effects, were performed when a significant interaction was detected. Partial eta squared (ηρ^2^) was used to indicate the effect size and to measure the main and interaction effects, where values of 0.04 = small, 0.25 = medium, and 0.64 = large effect size.^24^ The effect size of pairwise comparison was revealed by calculating Cohen's d (*d*), where *d* = 0.2, 0.5, and 0.8 indicate small, medium, and large effect sizes, respectively.^25^ Statistical signiﬁcance was set at *p* ≤ 0.05, and values are reported as means ± SD. The software of IBM SPSS Statistics 26 (IBM Corp., NY, US) was used for all the analyses.

## 3 Results

### 3.1 SIE performance

The peak power, mean power, and the fatigue index of the 30-s repeated sprints in SIE_2_ and SIE_4_ were shown in Table 2. During the repeated sprints, the mechanical load applied to the flywheel of the cycle ergometer was 4.76 ± 0.52 kp. The three SIE variables of the 1^st^ sprint bouts in the SIE_2_ and SIE_4_ were similar. The variables did not change significantly in the 2^nd^ bout in the SIE_2_. In contrast, the mean power, but not the other variables, of the 2^nd^ sprint bout in SIE_4_ was significantly lower than that of 1^st^ bout. In SIE_4_, significant decreases in the peak and mean power were further observed in the 3^rd^ and 4^th^ bouts. For the fatigue index, the first three bouts were similar, the 4^th^ bout was significantly lower than that of the 2^nd^ bout.

**Table 2:** The peak power, mean power, and the fatigue index of the 30-s repeated sprints in SIE_2_ and SIE_4_.

	SIE_2_		SIE_4_					SIE_2_*vs* SIE_4_
	1^st^ bout	2^nd^ bout	1^st^ bout	2^nd^ bout	3^rd^ bout	4^th^ bout	One-way ANOVA, *p* value (ηρ^2^)	2 × 2 ANOVA, *p* value (ηρ^2^)
Peak power (W)	452.2 ± 87.1	417.2 ± 65	483.9 ± 63.4	406.2 ± 71.6	343.1^a^^,^^b^ ±56.3	292.1^a^^,^^b^ ±65.2	*F*_(3)_ = 46 *p* < 0.001 (0.77)	Bouts: *F*_(1,14)_ = 20.1 *p* = 0.001 (0.59) Trials: *F*_(1,14)_ = 0.97 *p* = 0.34 (0.07) Interaction: *F*_(1,14)_ = 2.33 *p* = 0.15 (0.14)
Mean power (W)	380.6 ± 53.1	359.1 ± 58.8	409.5 ± 51.2	342.9^a^ ±63.2	299.7^a^^,^^b^ ±48.4	276.6^a^^,^^b^ ±41.5	*F*_(3)_ = 62.3 *p* < 0.001 (0.82)	Bouts: *F*_(1,14)_ = 16.9 *p* = 0.001 (0.55) Trials: *F*_(1,14)_ = 0.61 *p* = 0.45 (0.04) Interaction: *F*_(1,14)_ = 5.98 *p* = 0.03 (0.3)
Fatigue Index (%)	31.3 ± 14.4	33.7 ± 15.2	34.9 ±8.9	35.7 ±14	31.5 ± 14.8	21.6^b^ ±18	*F*_(3)_ = 5.53 *p* = 0.03 (0.28)	Bouts: *F*_(1,14)_ = 0.44 *p* = 0.52 (0.03) Trials: *F*_(1,14)_ = 1.17 *p* = 0.3 (0.08) Interaction: *F*_(1,14)_ = 0.19 *p* = 0.67 (0.01)

Trial abbreviations refer to the text.

a Significant different from 1^st^ sbout.

b Significant different from 2^nd^ bout.

### 3.2 HRV recovery

**Fig. 1:**
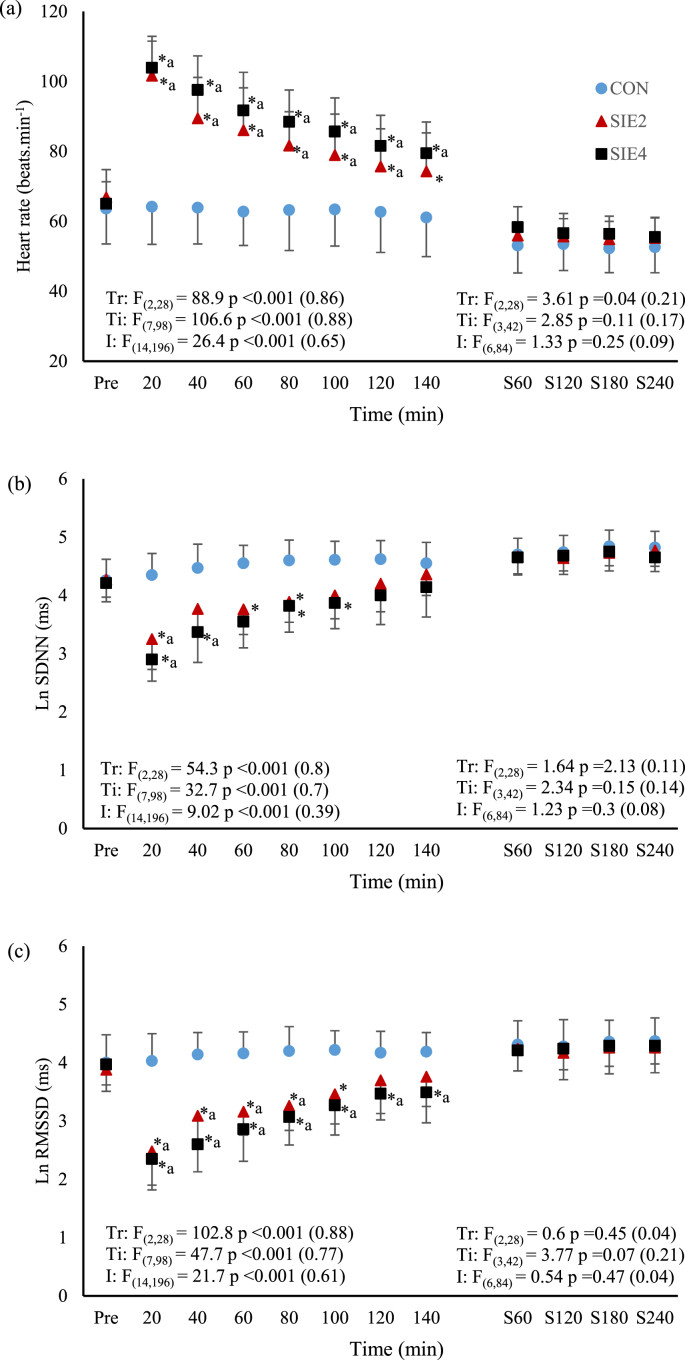
The time courses of (a) heart rate, (b) log standard deviation of normal to normal intervals (LnSDNN), and (c) log root mean square of successive R-R differences (LnRMSSD) before (Pre) and after the exercise, and during the first 4 h of actual sleep time in CON, SIE_2_, and SIE_4_ trials. Trial abbreviations refer to the text. ∗ significant different from corresponding CON value; ^a^ significant different from corresponding pre-exercise value.

For the time-domain HRV parameters, the results of SIE and CON trials are presented in Fig. 1a–c. Post-exercise HR in the SIE trials, but not CON trial, were higher than the corresponding pre-exercise value, except that at the time point of 140 min in SIE_2_ (*p* < 0.05, SIE_2_ *d* = 0.93–3.87; SIE_4_, *d* = 1.86–4.99). The HR following the repeated sprints in SIE_2_ and SIE_4_ were significantly higher than that of CON throughout the recovery period (*p* < 0.05, SIE_2_ *d* = 1.16–3.63; SIE_4_, *d* = 1.81–4.0). Although the HR in SIE_4_ appeared to be higher than that of SIE_2_, the differences were not significant (*p* > 0.05). For the LnSDNN, pre- and post-exercise CON values were not different (*p* > 0.05). Nevertheless, significant decreases from the corresponding pre-exercise levels (*p* < 0.05, SIE_2_ *d* = 2.4; SIE_4_, *d* = 1.69–3.79), as well as from the corresponding CON levels (*p* < 0.05, SIE_2_ *d* = 2.03–2.44; SIE_4_, *d* = 1.92–3.92), were found at selected time points in both SIE trials. The declined LnSDNN, in comparison to the corresponding CON levels, was regained starting from the time point of 100 min and 120 min, respectively, in SIE_2_ and SIE_4_. For the LnRMSSD, pre- and post-exercise CON values were also not different (*p* > 0.05). Following the SIE_2_, the index decreased from the pre-exercise (*p* < 0.05, *d* = 1.57–2.88) and CON (*p* < 0.05, *d* = 1.77–2.94) values until the time point of 80 and 100 min, respectively. In SIE_4_, the LnRMSSD throughout the recovery period were significantly lower than the pre-exercise (*p* < 0.05, *d* = 1.08–3.61), as well as the CON (*p* < 0.05, *d* = 1.61–3.35) values. The index between the SIE_2_ and SIE_4_ were not different (*p* > 0.05).

**Fig. 2:**
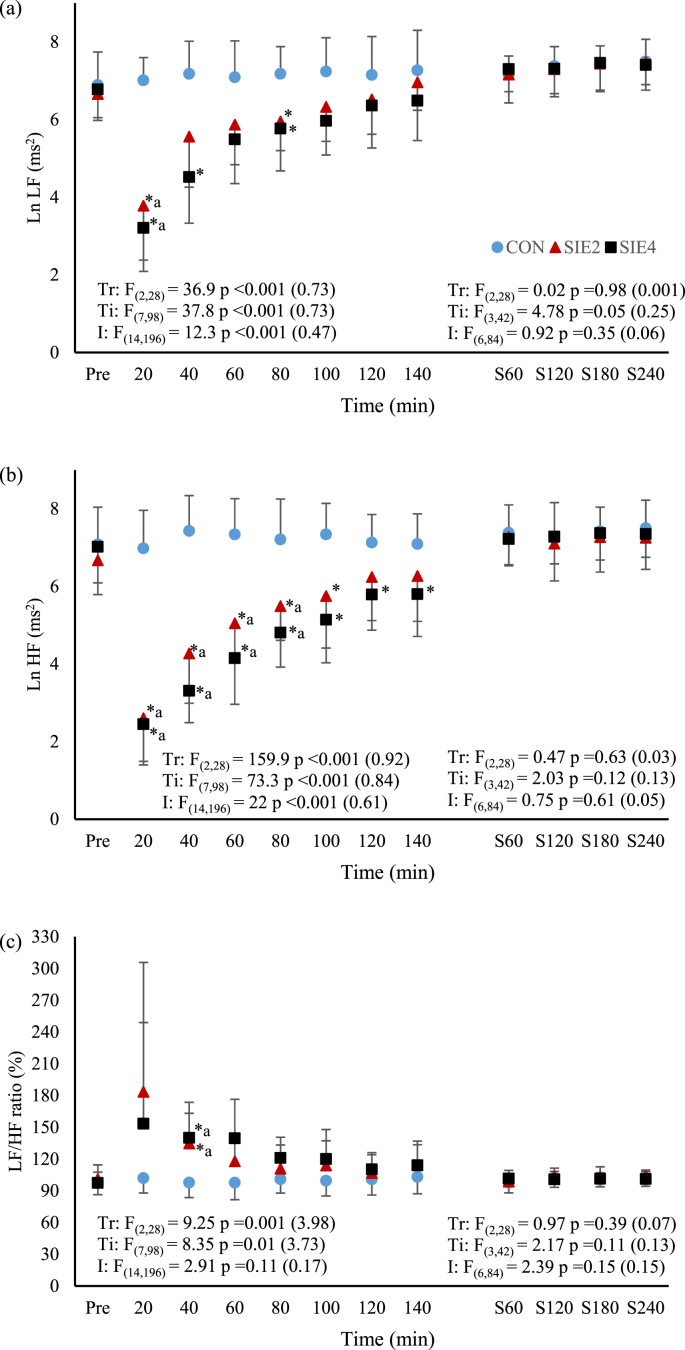
The time courses of (a) log low frequency (LnLF), (b) log high frequency (LnHF), and (c) LF/HF ratio before (Pre) and after the exercise, and during the first 4 h of actual sleep time in CON, SIE_2_, and SIE_4_ trials. Trial abbreviations refer to the text. The expressions of symbols for statistical significance refer to Fig. 1.

For the frequency domain indices of HRV (Fig. 2a–c), pre- and post-exercise CON values were not different (*p* > 0.05). In both SIE_2_ and SIE_4_ trials, LnLF at the time point of 20 min, and LnHF at the time points before 100 min decreased from the corresponding pre-exercise levels, while LF/HF ratio at the time point of 40 min increased from it (*p* < 0.05, LnLF: SIE_2_ *d* = 2.62; SIE_4_ *d* = 3.78, LnHF: SIE_2_ *d* = 1.34–3.87; SIE_4_ *d* = 2.43–4.84, LF/HF ratio: SIE_2_ *d* = 1.51; SIE_4_ *d* = 1.72). The LnLF at the time points of 20 and 80 min in SIE_2_, and at the time points of 20, 40 and 80 min in SIE_4_, were significantly lower than the corresponding CON values (*p* < 0.05, SIE_2_ *d* = 3.01 & 1.7; SIE_4_ *d* = 1.54–4.25). Significant attenuation of LnHF was also found in the SIE_2_ until the time point of 100 min (*p* < 0.05, *d* = 1.44–4.0), and in the SIE_4_ throughout the recovery period (*p* < 0.05, *d* = 1.36–4.76). The differences in LnLF and LnHF between the two SIE trials were not significant (*p* > 0.05). For the LF/HF ratio, it was significantly higher at the time point of 40 min in SIE_2_ and SIE_4_ in comparison to the corresponding CON value (*p* < 0.05, *d* = 1.64 in both trials), others were not different (*p* > 0.05). The ratio between the SIE_2_ and SIE_4_ was also not different (*p* > 0.05).

Among the three trials, the duration between exercise cessation and beginning of actual sleep were not different among the SIE and CON trials (SIE_2_: 6.2 ± 1.4, SIE_4_: 5.8 ± 1.1, CON: 6.0 ± 1.2 h, *p* > 0.05). Similar results were also found in the actual sleep time (SIE_2_: 7.3 ± 1.1, SIE_4_: 7.4 ± 1.8, CON: 6.8 ± 1.5 h, *p* > 0.05), and in the duration between the exercise cessation and the end of the nocturnal HRV measurements which was taken place in the first 4 h of actual sleep time (SIE_2_: 10.2 ± 1.4, SIE_4_: 9.8 ± 1.1, CON: 10.0 ± 1.2 h, *p* > 0.05). The nocturnal HRV parameters in SIE_2_, SIE_4_, and CON are shown in Fig. 1, Fig. 2 a - c. For the HRV indices measured during the first 4 h of the actual sleep time, the HR, LnSDNN, LnRMSSD, LnLF, LnHF, and LF/HF ratio, were not different among the time points and across the three trials (*p* > 0.05).

### 3.3 BP changes

**Fig. 3:**
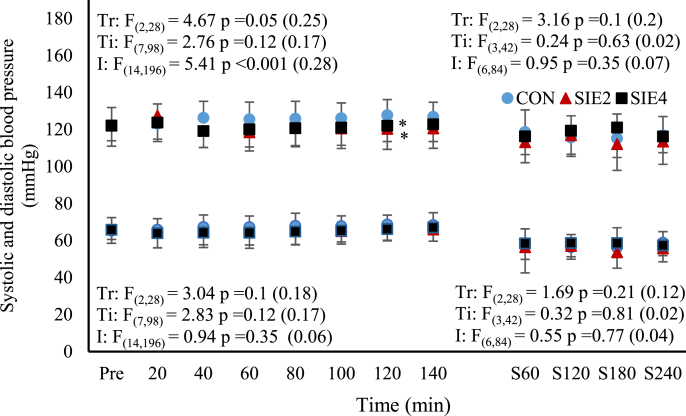
The time courses of systolic and diastolic blood pressure before (Pre) and after the exercise, and during the first 4 h of actual sleep time in CON, SIE_2_, and SIE_4_ trials. Trial abbreviations refer to the text. The expressions of symbols for statistical significance refer to Fig. 1.

Fig. 3 shows the time course of BP in the SIE and CON trials. The systolic and diastolic BP before and after exercise were not different in all trials (*p* > 0.05). Although the systolic BP in the SIE trials appeared to be lower than those at the corresponding time points in CON, significant difference was only found at the time point of 120 min in both SIE_2_ and SIE_4_ trials (*p* < 0.05, *d* = 0.74 & 0.68). For the diastolic BP, there was no significant difference among the time points and across the trials (*p* > 0.05). The nocturnal blood pressure among the three trials were also not different (*p* > 0.05).

## 4 Discussion

This study examined the HRV recovery following the SIE_2_ and SIE_4_, which the number of sprints of the SIE_4_ was twice as that of SIE_2_, by comparing with that of CON at corresponding time points. Essentially, it was found that the 30-s SIE in the SIE_2_ and SIE_4_ trials attenuated the HRV markedly, while the declined HRV restored progressively after the cessation of the exercise. Although the decline of the HRV indices in SIE_4_, mainly the LnRMSSD and LnHF, appeared to be greater than that in SIE_2_ during the recovery period, the differences between the two trials at any corresponding time point did not achieve statistical significance. Nevertheless, it appeared that the restoration of HRV in the SIE_2_ was in a faster rate in comparison to that of SIE_4_. As shown by the unvaried post-exercise nocturnal HRV at immediate night among the SIE and CON trials, the declined HRV following both SIE_2_ and SIE_4_ protocols were well restored after ∼10 h, irrespectively.

Exercise-induced HRV attenuation, as a function of exercise intensity, has been well demonstrated previously regardless of whether the HRV metric was based on time- or frequency-domain measures.^7^ The decay profile of HRV is curvilinear in nature; decreasing considerably with increasing exercise intensity, up to a particular level after which the declined HRV remains with no further substantial change.^7^ The intensity at which the HRV indices of cardiac parasympathetic neural activity, such as RMSSD and HF, reach minimum (HRV threshold) appears to be associated with that corresponding to the first ventilatory threshold.^26^, ^27^ In this study, the immediate changes of HRV in response to the SIE were not recorded. Nonetheless, the intensity of the sprints performed in both SIE_2_ and SIE_4_ were greatly higher than the first ventilatory threshold, allowing us to assume that the SIE-induced HRV attenuation were in similar magnitude in the two trials; the sympathetic-parasympathetic interactions elicited by the SIE_2_ and SIE_4_ protocols might not be markedly varied. This might partly explain the similar declines in LnRMSSD and LnHF recorded at the first time point of 20th min post exercise in the two trials (Fig. 1, Fig. 2b). In fact, apart from the two declined indices, the index of LnSDNN of overall HRV, and that of LnLF of both sympathetic and parasympathetic outflows also decreased significantly following the exercise, and the magnitudes of the decreases at the first time point post exercise were also similar between the two trials. However, the decrease, rather than increase, in the post-exercise LnLF, and the absence of marked increase in the LF/HF ratio, did not fully support the increased cardiac sympathetic modulation during the exercise.^28^ Nevertheless, HRV that quantifies the fluctuations in R-R intervals is basically used to monitor autonomic activity, particularly the cardiac parasympathetic modulation.^7^ Despite the LF of HRV is often used as an accurate reflection of cardiac sympathetic modulation in exercise and myocardial ischemia, the reciprocal change in parasympathetic activity appears to be a stronger influence in the LF power.^29^ Indeed, a decrease in LF during exercise was frequently reported in previous studies.^22^^,^^30^^,^^31^

Within 2 h after the SIE_2_ and SIE_4_ protocols, the declined HRV restored progressively (Fig. 1, Fig. 2). In agreement with the previous notion that exercise intensity, in comparison to other exercise variables, such as duration and modality, dominates the HRV attenuation during acute exercise as well as subsequent recovery,^7^ the declined HRV of each time point during the recovery were not significant different between the two trials despite of double of exercise bouts performed in SIE_4_. Similar findings have been reported previously after continuous exercise at light to moderate-high intensity.^7^ Nevertheless, we noted that the return of the declined LnSDNN, LnRMSSD and LnHF toward the CON level in SIE_4_ appeared in a rate slower than that of SIE_2_ (Fig. 1, Fig. 2). These suggest that the rate of removal of the cardiac autonomic disturbance, mainly revealed by the reactivation of parasympathetic activity in heart, following all-out sprint-interval exercise may be interfered by the exercise volume. However, the present data could not clearly elucidate the underlying mechanism for the delayed HRV recovery. It has been demonstrated that rather than the mean aerobic power or the net energy expenditure, the anaerobic process participation and associated heightened sympathetic activity and metaboreflex stimulation, such as muscle and blood acidosis, during repeated-sprint exercise primarily determine the level of post-exercise parasympathetic reactivation.^15^^,^^17^ Although we did not assess the metabolic stress resulting from the SIE_2_ and SIE_4_, it is reasonable to assume that the additional two bouts of 30-s sprints in SIE_4_ had possibly augmented the plasma norepinephrine levels as well as the metabolite persistence post exercise.^17^^,^^32^ The possible augmented sympathetic and metaboreflex stimulations might have caused interferences to the parasympathetic reactivation after the exercise in SIE_4_.^15^

Besides, high-intensity sprint-interval exercise has been reported to induce acute post-exercise plasma volume changes, a transient hypovolemia followed by hypervolemia.^33^^,^^34^ The associated baroreflex stimulation is likely to participate in determining the parasympathetic reactivation in the intermediate term (1–48 h) after the supramaximal exercise.^35^ It is not known if the changes in plasma volume following the SIE_2_ and SIE_4_ were varied due to the different exercise volume. Nevertheless, we noted that, in comparison to the CON level, there was a tiny but significant decrease in the post-exercise systolic BP in the SIE_2_ and SIE_4_ trials, with no difference between the two trials. The decrease in the BP was not observed during the actual sleep time period. It has been shown that the decrease in BP following exercise, termed as post-exercise hypotension, appears to be the mechanism for a gain of intravascular albumin via the lymph return, which promotes hypervolemia.^36^ Accordingly, the mild and comparable BP decrease, in addition of the unchanged nocturnal BP, following the SIE_2_ and SIE_4_ suggest that the plasma volume changes elicited by the two SIE protocols might not be markedly distinct, and it was not likely to contribute significantly to the varied vagal reactivation.

Our current data of HRV recovery following the SIE_2_ and SIE_4_ are in accordance with the previous findings that the time required for accomplishing recovery of suppressed cardiac vagal activity following two consecutive Wingate tests exceeded an hour.^22^ Further, based on our nocturnal HRV data (Fig. 1, Fig. 2), we found that the suppression of cardiac parasympathetic activity induced by the two SIE protocols were fully restored within a resting period of approximately 10 h after the cessation of the exercise (the duration between the exercise cessation and the end of the nocturnal HRV measurements). However, it has been reported that restoration of vagal activity precedes normalization of sympathetic cardiac nerves activity during later stage of post-exercise recovery.^22^, ^37^ Such scenario has been observed in the recovery of SIE_2_ (time points of 120th and 140th min) with which elevation of post-exercise HR was concomitant with the LnRMSSD and LnHF that were already restored to CON levels (Fig. 1, Fig. 2). The restored HRV after exercise concomitant with elevated HR suggests that the cardiovascular functions might be still disturbed.^22^^,^^37^ Nevertheless, all the indices of the nocturnal HR, BP, and HRV measured during the night sleep following the SIE_2_ and SIE_4_ cessations had restored well to CON levels. Based on these findings, it appears that the exercise volume of the all-out SIE protocol is not likely to be a predominant factor in attenuating cardiac parasympathetic activity. Nonetheless, delay of post-exercise vagal restoration resulting from the greater exercise volume of SIE_4_ was apparent. Irrespectively, a resting period of ∼10 h subsequent to either of the two SIE protocols could remove the exercise-induced cardiac autonomic disturbances, allowing the cardiovascular system to fully recover before engaging in next exercise session.

In the present study, there are some limitations deserve discussion. As the vagally related HRV measures is in synchrony with respiration,^38^ the respiratory rate of our participants during the HRV measurements was controlled at 12 breaths.min^−1^. The control of the respiratory rate might have perturbed the natural return of the post-exercise HR to baseline and diminished the external validity of the HRV results.^17^ Nevertheless, the identical respiratory rate during HRV measurements could minimize the potential discrepancy in the influences of the respiration on the HRV indices among the three trials. Another limitation is that only male participants were recruited in this study. The present findings of HRV in response to the SIE protocols in untrained young men should not be generalized for either trained, elderly, or female individuals.^8^^,^^39^ Further, the exercise-induced changes in the hormones or metabolites were not measured in this study, so the mechanisms elucidating our results are postulated. Besides, rebound of post-exercise cardiac parasympathetic activity above pre-exercise levels, partly attributed to the post-exercise hypervolemia, has been observed in the hours or days after exercise, and was considered as the optimal training period for attaining cardiorespiratory adaptations.^35^^,^^40^ However, the current study did not measure the intermediate HRV recovery (48 h post-exercise), limiting the comparison of the overcompensation of the parasympathetic reactivation between the two SIE protocols. Related measurements are suggested to be included in future studies. Finally, as nocturnal HRV was measured in a home environment, the time between the exercise cessation and the nocturnal HRV measurement were not uniform among participants, with variation of within 1 h. Moreover, their sleep environments were different. Nevertheless, all the exercise tests were scheduled at 19:00, and the analyzed 4-hr period for nocturnal HRV that occurred from about 6 h after the exercise cessation, was not likely to cause notable effect on the results. Indeed, the nocturnal HRV measurement taken place in participants' own home could largely reduce the disturbances caused by unaccustomed sleep place.

In conclusion, SIE_2_ and SIE_4_ protocols impaired the HRV in untrained young males in a similar magnitude although the repeated bouts of 30-s sprint in SIE_4_ was twice as that of SIE_2_. Nevertheless, the rate of HRV restoration appeared to be slower in SIE_4_. Regardless, the attenuation of HRV, and the concomitant changes in HR and BP, in both SIE_2_ and SIE_4_ trials after exercise cessation were well recovered within ∼10 h. In practice, the present findings provide updated guidelines for individuals who involve in prescription of sprint-interval training.^12^, ^13^, ^14^ Based on our findings, the time frame of ∼10 h appear to be appropriate for the cardiovascular system to recover between two sessions of the SIE. However, one should be aware that other systems, such as musculoskeletal or metabolic, which also work heavily during the exercise might not be recovered so soon and may depend upon one's training status and associated fitness level.

## Authors statement

Study conception and design: JN, ZK, XY; Acquisition of data: ZK, JN; Analysis and interpretation of the data: JN, ZK, TKT, YY; Drafting the paper: YY, TKT, EDT; Critical revision: YY, TKT, JN, XY; All authors read and approved the final version of the manuscript.

## Declaration of competing interest

All the authors declare that they have no conflict of interest.
